# The Canadian Pain Society: A historical perspective

**DOI:** 10.1080/24740527.2020.1810002

**Published:** 2020-10-06

**Authors:** Guillaume Léonard, Manon Choinière, Gilles Lavigne, Barry J. Sessle

**Affiliations:** a École de réadaptation, Faculté de médecine et des sciences de la santé, Université de Sherbrooke, Centre de recherche sur le vieillissement du Centre intégré universitaire de santé et de services sociaux de l’Estrie–Centre hospitalier universitaire de Sherbrooke, Sherbrooke, Canada; b Département d’anesthésiologie et de médecine de la douleur, Faculté de médecine, Université de Montréal, Centre de recherche du Centre hospitalier de l’Université de Montréal, Montreal, Canada; c Faculté de médecine dentaire, Université de Montréal, Centres de Recherche du Centre Hospitalier de l’Université de Montréal du CIUSSS Nord de l’Île de Montréal, Montreal, Canada; d Faculty of Dentistry, Department of Physiology, Faculty of Medicine, and Centre for the Study of Pain, University of Toronto, Toronto, Ontario, Canada

**Keywords:** Canada, pain, society, history, research, education, management

## Abstract

This article reviews the major features and events that have characterized the 40-year history of the Canadian Pain Society/Société canadienne de la douleur, which is a chapter of the International Association for the Study of Pain (IASP). The review first describes its early formative years in the 1970s as eastern and western chapters of IASP and then its evolution as a Canada-wide chapter and society. Also highlighted is the formulation in this period of its purpose to foster pain research, education, and management and the many activities in which the Society has been engaged to reinforce this purpose over the ensuing decades. These include its annual scientific meetings and the establishment of publications, guidelines, and other educational material as well as awards to support research and trainees. Many of these activities have included engagement with key partners who have also collaborated with the Society in national and international advocacy for pain. The review also outlines some of the features and factors underpinning the Society’s national and international reputation and impact resulting from the many contributions that its members have made to the advances in pain research, education, and management over the past 40 years. The review concludes by noting that by way of its rich history and its past and present experiences, the Society is well positioned to continue its many activities and contributions to address the many challenges still facing the pain field in Canada and around the world.

## RÉSUMÉ

Cet article passe en revue les principales caractéristiques et les principaux événements qui ont marqué les 40 ans d’histoire de la Société canadienne de la douleur, un chapitre de l’International Association pour l’étude de la douleur (IASP). On y décrit d’abord ses premières années de formation dans les années 1970 en tant que chapitres de l’Est et de l’Ouest de l’IASP, puis son évolution en tant que chapitre pancanadien et société. La formulation de son objectif, celui de favoriser la recherche sur la douleur, l’éducation à l’égard de celle-ci et sa prise en charge, ainsi que les nombreuses activités dans lesquelles la Société s’est engagée afin de renforcer cet objectif au cours des décennies suivantes, sont aussi mises en relief. Parmi celles-ci, on compte les réunions scientifiques annuelles et l’établissement de publications, de directives et d’autres matériels éducatifs, ainsi que de prix pour soutenir la recherche et les stagiaires. Bon nombre de ces activités comprenaient un engagement avec des partenaires qui ont également collaboré avec la Société dans le cadre de ses efforts de plaidoyer pour la douleur à l’échelle nationale et internationale. L’examen décrit également certaines des caractéristiques et des facteurs qui sous-tendent l’impact et la réputation internationale de la Société qui découlent des nombreuses contributions que ses membres ont apportées aux avancées en matière de recherche, à l’éducation sur la douleur et à la prise en charge de celle-ci au cours des 40 dernières années. La revue conclut en affirmant que par sa riche histoire et ses expériences passées et présentes, la Société est bien placée pour poursuivre ses nombreuses activités et continuer à apporter sa contribution afin de répondre aux nombreux défis auxquels le domaine de la douleur est encore confronté au Canada et dans le monde.

## The Formative Years

The Canadian Pain Society/Société canadienne de la douleur is a chapter of the International Association for the Study of Pain (IASP). Founded in 1973, IASP held its first international conference on pain in 1975, in Florence, Italy.^1^ Denise Able-Fessard, professor and researcher at Université de Paris (France), was chair of the Scientific Program Committee for this First World Congress on Pain of the IASP. She was also elected as first president of IASP.

The First World Congress on Pain was a key event in the early history of the Canadian Pain Society. It is clear from Merskey’s detailed outline of the history of pain research and management in Canada up to the mid-1990s^2^ that many Canadian clinicians and researchers had been working in the field of pain up to the time of the First World Congress on Pain. Several of them attended this congress, including Richard Catchlove from McGill University, who took the initiative at the congress to obtain sufficient signatures from Canadian members to petition IASP for the creation of the Eastern Canadian Chapter of IASP. This request was approved at the General Assembly held during the congress, and Dr. Catchlove was elected as the first president of the Eastern Canadian chapter (see Table 1).

**Table 1.** Chronological list of presidents of the Canadian Pain Society with their professional or scientific field and interests in the study of pain

1976–1979	Richard Catchlove, anesthesiologist; pharmacology and clinical research
1979–1982	Ramon Evans, surgeon; pain and cancer
1982–1985	Ian Purkis, anesthesiologist; pharmacology, emergency medicine, and traumatology
1985–1988	James Henry, physiologist and pharmacologist; pain mechanisms
1988–1991	Harold Merskey, psychiatrist; pain and vulnerable patients
1991–1994	Ronald Tasker, neurosurgeon; trigeminal pain and neuroma
1994–1997	Kenneth Craig, psychologist; pain and psychosocial factors
1997–2000	Alexander John Clark, anesthesiologist; management of peri-operative pain
2000–2003	Celeste Johnston, nurse; pediatric pain
2003–2005	Gary Rollman, psychologist; pain and psychosocial factors
2005–2007	Roman Jovey, family physician; pain and addiction
2007–2009	Barry Sessle, dentist and physiologist; oroacial pain, pain and sleep
2009–2011	Mary Lynch, psychiatrist; pain, opioids and cannabis
2011–2012	Catherine Bushnell, psychologist; pain and imagery
2012–2014	Judy Watt-Watson, nurse; postoperative pain, education
2014–2016	Gilles Lavigne, dentist and physiologist; oroacial and posttraumatic pain, pain and sleep
2016–2018	Brian Cairns, pharmacist and pharmacologist; temporomandibular pain
2018–2020	Fiona Campbell, anesthesiologist; pediatric pain
2020–2022	Karen Davis, physiologist; pain and neuroimaging

Building on the IASP’s momentum and the enthusiasm of the members, the Eastern Canadian chapter held its first annual scientific meeting in Montreal in 1976. Montreal was also the host city for IASP’s Second World Congress on Pain that was held two years later. John Liebeskind, from the University of California in Los Angeles, was the chair of the Scientific Program Committee and Ronald Melzack, from McGill University, was chair of the Local Arrangements Committee.

This was a particularly lively period in Quebec and the rest of Canada, with cultural blossoming and major international events such as Expo 67 and later the 1976 Olympic Games that were hosted by Montreal. These activities led to Montreal and Canada gaining increased attention on the world stage. Also notable was the location of the Second World Congress on Pain in 1978; it was held in the historic Queen Elizabeth Hotel located in downtown Montreal. This hotel received many prominent figures of the 20th century, such as Fidel Castro, Jacques Chirac, the Dalai Lama (Tenzin Gyatso), Charles de Gaulle, Indira Gandhi, Mikhail Gorbachev, Henry Kissinger, and Nelson Mandela. In 1969, the hotel also welcomed John Lennon and Yoko Ono, who, after being refused entry to the United States, had to stay there for an extended period of time, during which occurred the famous “Bed-In” in room 1742 of the hotel in which they wrote and recorded the song “Give Peace a Chance.”

By the time of the Second World Congress on Pain in Montreal, a Western Canadian chapter had also been established. The meetings and events in Montreal, including the mythical song by Lennon and Ono, undoubtedly influenced the destiny of pain research and management of pain in Canada. They were factors that awakened our thinking, encouraging both Canadian chapters of the IASP to join forces and petition IASP for the formation of a Canada-wide IASP chapter. The establishment of the chapter was approved by the IASP council, and Richard Catchlove was elected as its first president. Under Dr. Catchlove’s leadership, its constitution and bylaws were formulated and subsequently approved in 1980 during the first annual scientific meeting of the Canadian chapter that was held in Montreal.

## From a Chapter to One Encompassing a Society/Société

In 1983, driven by the chapter President Ian Purkis (Dalhousie University, Nova Scotia), Past-President Ramon Evans (University of Toronto), and Secretary Barry Sessle (University of Toronto), the chapter’s name was formally changed to The Canadian Pain Society—A Chapter of the International Association for the Study of Pain, with appropriate modifications to the chapter’s bylaws. Subsequent significant amendments to the bylaws were made in 1985 and 2012, and the Society was fully incorporated in 2006. The amendments included recognition of the name of the Society in French, in order to reflect our country’s cultural and linguistic reality. Since then, the Society has been known as the Canadian Pain Society/Société canadienne de la douleur.

## Reinforcement of the Society’s Purpose

The Canadian Pain Society/Société canadienne de la douleur has kept the purpose outlined in the earlier formulations of the chapter, namely, “To foster and encourage research on pain mechanisms and pain syndromes and to help improve the management of patients with acute and chronic pain by bringing together the basic scientists and health professionals of various disciplines and backgrounds who have an interest in pain research and management.” (Society Bylaws, page 17). Since its early days as an IASP chapter, membership of the Society has grown from around 100 (in the 1970s) to almost 1000 members by 2010 when the World Congress on Pain was held again in Canada. For many years, membership categories included regular, affiliate, trainee, corporate, life, and honorary members. Nowadays, the categories are regular, trainee, life, retired, and honorary members. The current membership stands at around 700. All clinical disciplines related to pain management are represented in the membership of the Society, including medicine, dentistry, nursing, psychology, sociology, chiropractic, pharmacy, veterinary medicine, and physiotherapy. In addition, several medical specialties (e.g., anesthesiology, neurology, neurosurgery, pediatrics, psychiatry, rehabilitative medicine) and many basic sciences (e.g., biochemistry, physiology, pharmacology, neurosciences) are represented. The Society’s all-inclusive and multidisciplinary nature is also reflected in the line of the presidents shown in Table 1.

The Society’s mandate requires it to be involved in a variety of activities to foster pain research, management, and education. Though these activities have expanded since its early years, the marquee event is still the annual scientific meeting, which has been held in various cities across Canada since the late 1970s. The annual scientific meeting of the Society continues to be a wonderful opportunity for researchers, clinicians, trainees, patient representatives, and industry partners with an interest in the area of pain to meet and share research findings, clinical approaches, and new ideas, as well as engage in social activities. Sadly, the 2020 annual scientific meeting had to be canceled because of the COVID-19 pandemic, but the Society was able to innovate and in a sense reinvent itself, offering a series of webinars and other online educational activities to its members.

Most of the annual scientific meetings for the past 40 years have been solely operated by the Society, but two have been conjoint meetings with the American Pain Society, held in Toronto in 1988 and in Vancouver in 2004, and two others have been conjoint meetings with the British Pain Society, held in Halifax in 1985 and in Edinburgh in 2011 (or its forerunner, the Intractable Pain Society of Great Britain and Ireland).

The Society has also been involved in other meetings, most notable being its major role in the IASP World Congresses on Pain, not only the 1978 congress (see above) but also two subsequent congresses that took place in Vancouver in 1996 and again in Montreal in 2010; Society members such as Kenneth Craig (University of British Columbia), Jeffrey Mogil (McGill University) and Manon Choinière (Université de Montréal) were instrumental in the organization of these congresses. Another World Congress on Pain is scheduled for Canada: the 2022 World Congress on Pain is to be held in Toronto.

## Establishment of Publications, Guidelines, and Other Educational Material for the Pain Community

Another regular activity of the Society is the publication of several issues of its own journal. This started in 1996 when, with the external publishing firm Pulsus, the Society launched *Pain Research and Management*, under the editorial direction of Harold Merskey, who was subsequently succeeded by Kenneth Craig. Then, after 20 years, the Society changed publishers and established the *Canadian Journal of Pain/Revue canadienne de la douleur*, with Joel Katz as Editor-in-Chief and the Taylor & Francis Group as publisher; the first issue of the new journal was published in 2017. Through the initiative of President Jim Henry in 1986, the Society also established a regular newsletter and the logo of the Society, and since then the newsletter bearing this logo has been circulated several times each year to inform members of Society events, activities, awards, grants, etc.

For the past 20 years or so, the Society has been involved in several other activities promoting pain research, education, and improved pain management and care. These include the creation of three special interest groups (SIGs; Interventional Pain, Neuropathic Pain, and Interprofessional Pain Management). These SIGs usually come together during the annual scientific meeting of the Society and have activities engaging their members throughout the year. Another example is the Society’s long-held commitment to supporting trainees and young investigators, whether through travel grants for trainees to participate in meetings or through awards of research funds to young researchers. Prestigious awards are also made each year to recognize individuals who have had a significant impact on pain research, education, management, or advocacy. Several of these awards and grants over the years have been supported in part by sponsors and partners of the Society.

Another notable activity of the Society during the past three decades has been the development of fact sheets, guidelines, and position statements, such as that in 2015 on the prevention of the herpes zoster virus^3^ and another in 2018 on the use of opioid analgesics in pain management.^4^ Society members have also contributed to guidelines and position statements developed by the IASP (e.g., access to care for pain; pain education). A particularly notable activity in this regard was that associated with the 2010 World Congress on Pain held in Montreal. The international Pain Summit took place at the end of the Montreal congress, and Society members played key roles in organizing the summit, which led to the Declaration of Montreal,^5^ which embraced the following three articles:

*Article 1*. The right of all people to have access to pain management without discrimination.

*Article 2*. The right of people in pain to acknowledgment of their pain and to be informed about how it can be assessed and managed.

*Article 3*. The right of all people with pain to have access to appropriate assessment and treatment of the pain by adequately trained health care professionals. (page 1)

## Interactions with Key Partners in Canada

The Society has a long history of working with patients through various pain advocacy groups such as the Canadian Pain Coalition (CPC), the Chronic Pain Association of Canada, Association québécoise de douleur chronique, and Pain BC. The Pain Summit in Montreal mentioned above is one example; here the Society collaborated in particular with CPC and Quebec partners. Another is the Society’s partnering with CPC, which led to the establishment 16 years ago of the National Pain Awareness Week, which was approved by the Canadian Senate in 2004 and takes place each year to raise the awareness of the public, media, and policymakers about pain and the impacts of chronic pain. Society members also established a Canadian Pain Foundation that was incorporated in 1983 as a charitable organization to provide fiscal support for pain research and education initiatives. The Foundation assisted in the development of the Society, but insufficient funding was raised over the last two to three decades and it was discontinued. Collaborations with research networks and government agencies have also been created; for example, Réseau québécois de recherche sur la douleur du Fonds de recherche du Québec–Santé, the Chronic Pain Network of the Canadian Institutes of Health Research, Research Canada (including the Health Research Caucus on Pain^6^), and the Canadian Pain Task Force associated with Health Canada.

The Canadian Pain Task Force is especially noteworthy given the decades spent by the Society and its patient partners and others in lobbying governments and policymakers to raise pain awareness and address the pain crisis in Canada. In 2018, in the wake of the North American opioid crisis, several Society members took part in a round table discussion with Canada’s minister of health to address the management of chronic pain in this country. This historic meeting led to the creation in 2019 of the Canadian Pain Task Force, as an initiative of Health Canada. The official announcement of the task force, which includes several members of the Society, was made at the Society’s annual scientific meeting in Toronto in 2019 by the minister, who noted that the task force’s mandate is to counsel politicians and decision makers in the implementation of improved approaches to prevent and manage chronic pain in Canada.^7^ It is hoped that this historic initiative will produce meaningful advances in pain management, education, and research in Canada and become a landmark feature of the history of the Society.

## Factors Underpinning the Society’s National and International Reputation and Impact

The high national and international profile of the Society has been bolstered by objective evidence that Canada is a world leader in research, clinical, and educational initiatives on pain. Many members of the Society have acquired an international reputation for their innovative research and clinical approaches. This can be particularly seen in the quality and impact of their publications: 4 of the 11 most cited articles on pain worldwide were published by Canadian researchers from the University of Toronto and McGill University, two institutions that are also among the top 16 universities with the highest outreach impact in the world.^8^ Canadian researchers and institutions also stand out in specific pain areas. For example, Canada ranks among the top 10 countries for the number of cited articles on research on low back pain (with the University of Alberta being the lead university), and Canada is among the top five countries for research on neuropathic pain (with McGill University and the University of Toronto being among the leading universities in this field).^9–11^ We must also mention that Society members are recognized world leaders in other pain areas, such as orofacial pain, pain genetics, and pediatric pain.^2,12,13^ And we must not forget the numerous books related to pain that have been published by IASP Press and other publishers and that have been edited or authored by members of the Society on subjects as varied as the classification of pain, neuropathic pain, orofacial pain, pediatric pain, pain and sleep interactions, genetics, pharmacology, psychology, and aging.

The international impact of Canada and the Society can also be seen through the contributions of its members to IASP itself, whether in the roles played by Ronald Melzack, Barry Sessle, and Fernando Cervero as presidents of this prestigious international organization or in the responsibilities taken on by other individuals such as Eduardo Bruera, Catherine Bushnell, Eloise Carr, Karen Davis, Jonathan Dostrovsky, Allan Finley, Ian Gilron, Mary Ellen Jeans, David Lussier, Mary Lynch, Patricia McGrath, Patrick McGrath, Jeffrey Mogil, Jennifer Stinson, Ronald Tasker, and Judy Watt-Watson, who have served in various other roles for IASP (e.g., IASP council member, chair of an IASP SIG or task force, or member of an editorial committee of the journal PAIN or other IASP publications).

## Concluding Remarks

It is clear from this historical perspective that the Canadian Pain Society/Société canadienne de la douleur has much to be proud of. Yet, despite its rich past and solid foundation and the many contributions that its members have made to the advances in the pain field over the past 40 years, several challenges still remain. These include the unmet needs stemming from the opioid crisis, of which we still feel the effects; the unknown long-term health consequences resulting from the legalization of cannabis in Canada; the slow advancement of innovative pain relief therapies based on scientific evidence; the loss of continuity of care for chronic pain; the multiplication of intersectoral services and care that go beyond traditional health care professions; the need for integration of personalized medicine into approaches for pain relief; problems with timely access to appropriate pain management by many Canadians, especially those living in rural or remote areas and/or having socioeconomic hardships; and, last but not least, the limited funding for pain research. All of these challenges are still present within a milieu of complex, interacting factors that include the following: (1) the approximately 7.5 million Canadians already suffering from chronic pain; (2) the future growth in this number as demographic changes result in a higher proportion of the population being elderly and suffering from chronic diseases, most of which also manifest pain; (3) the enormous socioeconomic costs of pain, which continue to rise; (4) and the need to improve the education of clinicians to enhance their knowledge base of pain and their ability to deal with the growing epidemic of pain. Clearly, the Society cannot rest on its laurels and still has a crucial role to play in the pain field in Canada and the rest of the world. Based on its history and its past and present experiences with fostering pain research, education, and management, the Society is well positioned to continue its many activities and contributions, which will be needed more than ever in the upcoming decades.
